# Improvement of maize drought tolerance by foliar application of zinc selenide quantum dots

**DOI:** 10.3389/fpls.2024.1478654

**Published:** 2024-12-03

**Authors:** Venkatesan Kishanth Kanna, Maduraimuthu Djanaguiraman, Alagarswamy Senthil, Ponnuraj Sathya Moorthy, Krishnamoorthy Iyanar, Anbazhagan Veerappan

**Affiliations:** ^1^ Department of Crop Physiology, Tamil Nadu Agricultural University, Coimbatore, India; ^2^ Department of Basic Engineering & Applied Sciences, Agricultural Engineering College & Research Institute, Kumulur, India; ^3^ Department of Millets, Tamil Nadu Agricultural University, Coimbatore, India; ^4^ Department of Chemistry, School of Chemical and Biotechnology, SASTRA Deemed University, Thanjavur, India

**Keywords:** maize, drought, nano-zinc composite, photosynthesis, stomatal conductance

## Abstract

Maize (*Zea mays* L.) is an important cereal crop grown in arid and semiarid regions of the world. During the reproductive phase, it is more frequently exposed to drought stress, resulting in lower grain yield due to oxidative damage. Selenium and zinc oxide nanoparticles possess inherent antioxidant properties that can alleviate drought-induced oxidative stress by the catalytic scavenging of reactive oxygen species, thereby protecting maize photosynthesis and grain yield. However, the effect of zinc selenide quantum dots (ZnSe QDs) under drought stress was not been quantified. Hence, the aim of this study was to quantify the (i) toxicity potential of ZnSe QDs and (ii) drought mitigation potential of ZnSe QDs by assessing the transpiration rate, photosynthetic rate, oxidant production, antioxidant enzyme activity and seed yield of maize under limited soil moisture levels. Toxicity experiments were carried out with 0 mg L^−1^ to 500 mg L^−1^ of ZnSe QDs on earthworms and azolla. The results showed that up to 20 mg L^−1^, the growth rates of earthworms and azolla were not affected. The dry-down experiment was conducted with three treatments: foliar spray of (i) water, (ii) ZnSe QDs (20 mg L^−1^), and (iii) combined zinc sulfate (10 mg L^−1^) and sodium selenate (10 mg L^−1^). ZnSe or Se applications under drying soil reduced the transpiration rate compared to water spray by partially closing the stomata. ZnSe application at 20 mg L^−1^ at the tasselling stage significantly increased the photosynthetic rate (25%) by increasing catalase (98%) and peroxidase (85%) enzyme activity and decreased the hydrogen peroxide (23%) content compared to water spray, indicating that premature leaf senescence was delayed under rainfed conditions. ZnSe spray increased seed yield (26%) over water spray by increasing the number of seeds cob^-1^ (42%). The study concluded that foliar application of ZnSe (20 mg L^−1^) could decrease drought-induced effects in maize.

## 1 Introduction

The global population is expected to reach 10.4 billion by 2023, of which 281.6 million people experienced food insecurity, and this will continue to be the case as the population continues to grow. To address this challenge, increasing crop productivity in stressful environments is one approach (Singh et al., 2023). Among the various abiotic stresses, drought is an important factor affecting crop productivity and is a serious problem due to climatic changes and the unpredictable and irregular distribution of rainfall patterns (Zeng et al., 2023). Therefore, it is crucial to develop drought-tolerant varieties and efficient crop management strategies to maintain productivity.

Globally, next to rice (*Oryza sativa* L.) and wheat (*Triticum aestivum* L.), maize (*Zea mays* L.) is the predominant crop grown for food, feed, and fuel. Globally, 50% of the maize cultivation areas are under rainfed conditions (Biradar et al., 2009). In the USA, 92% of the maize-growing region is rainfed (Kimm et al., 2020), and a similar situation has prevailed in China, sub-Saharan Africa, and India (Hu et al., 2023; Erenstein et al., 2022). Maize is more sensitive to drought stress than wheat (Kim and Lee, 2023). Drought stress during the vegetative, pollination, and grain-filling stages of maize reduced the grain yield by 25%, 50%, and 21%, respectively, suggesting that the reproductive phases are more sensitive than other growth stages (Sah et al., 2020). When drought stress occurs two weeks before anthesis, the yield decreases by 3%–4% per day (Nielsen, 2002). Yield decline depends on the intensity and duration of drought stress and crop stage. Based on weather and historical crop production data, rainfed maize grain yield in arid and semiarid regions is predicted to decline by 22% by 2050 (Schlenker and Lobell, 2010). In general, maize is more suitable for irrigated than rainfed ecosystems, as evidenced by the magnitude of the yield decrease due to differential rainfall patterns in the rainfed ecosystem (Qiao et al., 2021). Therefore, it is critical to develop a crop management strategy using an alternative approach to sustain the yield potential of rainfed maize.

Drought stress has diverse effects on crop growth, physiology, and grain yields. The major effects of drought stress are associated with the loss of photosynthetic activity, increased abscisic acid biosynthesis, improved osmotic adjustment, and improved antioxidant defense systems in maize (Sheoran et al., 2022; Gong et al., 2014). A reduction in photosynthesis may be associated with reduced stomatal conductance, which prevents water loss owing to reduced tissue turgor pressure and water potential (Singh and Reddy, 2011). Stomatal regulation is related to transpiration rate rather than photosynthetic rate because of high stomatal conductance, and photosynthetic rate levels off; however, the transpiration continues to rise linearly under mild drought stress (Condon et al., 2002; Djanaguiraman et al., 2024). However, the reduced photosynthetic rate is primarily due to stomatal limitation, which is evident from the decreased intercellular to atmospheric CO_2_ levels (C_i_/C_a_ ratio) under moderate drought stress (Flexas et al., 2002). A decrease in the photosynthetic rate is associated with reduced photochemistry and photosynthetic enzyme activity under severe drought stress (Aranjuelo et al., 2011; Edmeades et al., 1999).

Drought stress during the flowering stage results in a reduction in the number of grains per cob and lower grain yield in maize (Edmeades et al., 1999). Under severe drought stress, a reduced seed-set percentage is associated with loss of pollen and pistil functions (Boyer and Westgate, 2004), resulting in complete seed abortion (Li et al., 2023). Drought also increases the production of reactive oxygen species (ROS), such as superoxide radicals, hydrogen peroxide, and singlet oxygen, and excessive ROS production causes oxidative damage to cell organelles, especially chloroplasts, impairing the normal function of chloroplasts at the cellular level (Dietz and Pfannschmidt, 2011; Foyer and Noctor, 2009). The ability of the cell to scavenge the generated ROS depends on the activity of antioxidant enzymes, such as superoxide dismutase, catalase, and peroxidase (Faize et al., 2011; Yang et al., 2021). Foliar application of chemicals possessing antioxidant properties is an alternative approach for minimizing the effects of ROS under drought stress.

Recently, the use of nanoparticles with antioxidant properties to alleviate drought has increased (Djanaguiraman et al., 2024; Li et al., 2024). The use of nanomaterials to mitigate oxidative damage is more effective because of their exceptionally increased surface area (Omran and Baek, 2021; Xu et al., 2023). However, the antioxidant properties of nanomaterials differ depending on their chemical configuration, surface coating, charge, size, and crystallinity (Shah et al., 2017). The most commonly used metallic nanomaterials to alleviate oxidative damage are silver (Khan et al., 2020), ceria (Djanaguiraman et al., 2024), copper (Noman et al., 2020), copper oxide (Xiong et al., 2021), hematite (Al-Amri et al., 2020), selenium (Djanaguiraman et al., 2018), silica (Wang et al., 2021), silicon dioxide (Banerjee et al., 2021), titanium dioxide (Hu et al., 2020), and zinc oxide (Rai-Kalal and Jajoo, 2021). Overall, nano-zinc and nano-selenium possess antioxidant properties and can be used to mitigate drought-induced oxidative stress in plants. However, to our knowledge, the antioxidant properties of zinc selenide quantum dots (ZnSe QDs) under normal and drought stress conditions in plants have not yet been validated.

Hence, we hypothesized that the exogenous application of nanomaterials (ZnSe QDs) possessing antioxidant properties is an effective and convenient approach to mitigate drought-induced oxidative stress in maize. The aim of this study was to quantify the (i) toxicity potential of ZnSe QDs and (ii) drought mitigation potential of ZnSe QDs by assessing the transpiration rate, photosynthetic rate, oxidant production, antioxidant enzyme activity and seed yield of maize under limited soil moisture levels.

## 2 Materials and methods

### 2.1 Synthesis and characterization of ZnSe QDs

ZnSe QDs were synthesized using selenium metal and zinc chloride as precursors for selenium and zinc, respectively (Jayalakshmi et al., 2024). The shape, size, and purity of the formed ZnSe QDs were analyzed using a particle size analyzer, Fourier transform infrared (FTIR) spectroscopy, scanning electron microscope (SEM), transmission electron microscope (TEM), and high-resolution transmission electron microscope (HRTEM) as described by Djanaguiraman et al. (2018). Selected area electron diffraction (SAED) and energy-dispersive X-ray spectroscopy (EDAX) were performed using HRTEM. A diffractometer was used to analyze the powder X-ray diffraction (XRD) in the 2θ range of 20°–80° (Djanaguiraman et al., 2018).

### 2.2 Quantifying the toxicity of ZnSe QDs

#### 2.2.1 Earthworm

The earthworm (*Eisenia andrei*) was used as a test organism to quantify the toxic effects of ZnSe QDs (OECD, Test No. 207, 1984; Aderjan et al., 2023). Briefly, adult earthworms weighing 500 mg–600 mg were collected from natural clay soil and acclimated to the soil for 48 h. Adult earthworms with clitella were placed in a high-density polyethylene container (500 mL capacity) filled with 100 g soil. The clay soil was then dried, powdered, and sieved. The soil was moistened with ZnSe QDs solution to maintain its water-holding capacity. In this study, ten different concentrations of ZnSe QDs (0 mg L^−1^, 5 mg L^−1^, 10 mg L^−1^, 15 mg L^−1^, 20 mg L^−1^, 25 mg L^−1^, 50 mg L^−1^, 100 mg L^−1^, 200 mg L^−1^, and 500 mg L^−1^) were tested, with 10 replicates for each concentration. Earthworms were placed in polyethylene containers and maintained for 14 d. Earthworm mortality was recorded on days 7 and 14 after exposure, and the change in weight was recorded on the 14^th^ day. The immobilized earthworms were considered dead. The growth rate of the remaining earthworms was determined by dividing the fresh weight by 14 and was expressed as mg d^−1^.

#### 2.2.2 Azolla

Azolla sp. was used as a model organism to quantify the toxic effects of ZnSe QDs in aquatic species. Azolla sp. were grown in the International Rice Research Institute medium, as described by Pereira and Carrapiço (2009). Preweighed (500 mg) azolla was added to 1,000 mL of the medium with different concentrations of ZnSe QDs (0 mg L^−1^, 5 mg L^−1^, 10 mg L^−1^, 15 mg L^−1^, 20 mg L^−1^, 25 mg L^−1^, 50 mg L^−1^, 100 mg L^−1^, 200 mg L^−1^, and 500 mg L^−1^), with 10 replicates for each concentration. Azolla was grown for 15 d, collected from different concentrations and dried at 60 °C for 48 h to obtain the dry weight. The growth rate was determined by dividing the dry weight by 15 and was expressed in mg d^−1^.

### 2.3 Effect of ZnSe QDs on the transpiration rate in progressive soil drying

To validate the hypothesis that foliar application of ZnSe QDs during the peak vegetative stage can limit the transpiration rate under progressive soil drying, a factorial randomized block design experiment with five replications was conducted. The soil moisture regime was factor 1 with two levels [irrigated control: watered every evening to maintain 0.9 fraction of transpirable water (FTSW) and progressive soil drying: no irrigation from 50 days after emergence (DAE) till completion of the experiment], and foliar spray was the factor 2 with three levels [water, ZnSe QDs (20 mg L^−1^ of the ZnSe QDs), and bulk: b-Zn-Se (10 mg L^−1^ of ZnSO_4_ + 10 mg L^−1^ of sodium selenate)].

The soil (pH: 7.8, electrical conductivity: 0.24 dS m^−1^) from the Tamil Nadu Agricultural University, Coimbatore, India farm was transported to the glasshouse, dried, powdered, sieved and used. Co(H)M 8 seeds were planted in a plastic pot with 27 kg of clay loam soil. Two grams of urea, one gram of diammonium phosphate, and one gram of potash were added to each pot and repeated at 15 DAE, 30 DAE, and 45 DAE. The pots had holes at the bottom for drainage. Three seeds were planted at a depth of 3 cm each. One plant per pot was maintained after emergence. Weather data during the experimental period were monitored using the HOBO weather station (Onset Computer, Boume, Massachusetts, USA). The air temperature was measured using a shielded thermocouple 10 cm above the canopy.

The pots were watered every other day until the eighth leaf fully emerged (50 days after emergence; DAE). On the 51^st^ DAE, pots were divided into two groups: group 1 (irrigated control) and group 2 (progressive soil drying). At 6 p.m., all pots were watered to field capacity and covered with a polyvinyl chloride sheet to prevent evaporation. The following morning, eight pots in each group were sprayed with water. The other eight pots were sprayed with ZnSe QDs at a concentration of 20 mg L^−1^. The remaining eight pots were sprayed with bulk zinc and selenium (10 mg L^−1^ ZnSO_4_ and 10 mg L^−1^ sodium selenate) solution.

From 52 DAE to 65 DAE, all pots were weighed at 7 a.m. and 6 p.m. to record the fraction of transpirable soil water (FTSW). The FTSW was calculated according to Sinclair and Ludlow (1986). The amount of water lost during the daytime was added at 6 p.m. daily to maintain 0.9 FTSW levels for the irrigated pots. However, no water was added to the progressive drying pots until FTSW reached 0.08. The hourly transpiration rate was recorded from 8 a.m. to 6 p.m. continuously for 7 d and was used to calculate the difference between the two consecutive column weights and was expressed in g plant^−1^. Water use efficiency (WUE) was calculated as the ratio between the amount of weight gain and the amount of water added and was expressed in g L^−1^. The quantity of dry matter accumulated relative to the amount of water applied was used to calculate the WUE.

The surface of the boot leaf was cleaned with filter paper, and a thin layer of transparent nail polish was applied and allowed to dry. After 10 min, dried enamel was carefully removed using a surgical blade. The peel was kept in distilled water and visualized under a fluorescent microscope to record the stomatal aperture size and opening (DX 3153-APLi, Delphi-X, Arnhem, The Netherlands) at ×400 magnification.

### 2.4 Effect of ZnSe QDs under rainfed condition

The field experiment was conducted in a randomized block design with four replicates. The treatments included four foliar sprays: 1. water; 2. bulk: 10 mg L^−1^ ZnSO_4_ + 10 mg L^−1^ sodium selenate; 3. 20 mg L^−1^ ZnSe QDs; and 4. Zn-EDTA: 20 mg L^−1^ zinc-EDTA. Maize cultivar CoH(M) 8 was planted at a spacing of 60 × 30 cm, and the pre-emergence herbicide atrazine was sprayed. Life irrigation was given on the third DAP, and thinning was performed on the 10^th^ DAP. Plants were fertilized with 135:62.5:50 NPK kg ha^−1^, and the irrigation was given on the 14^th^ DAP. Plants were maintained under rainfed conditions. Rain was received on the 20^th^ and 27^th^ DAP with 1.2 mm and 4.3 mm, respectively, followed by one final irrigation given on the 40^th^ DAP (at the active vegetative stage). Foliar spray treatments were imposed on the 48^th^ DAS. The weather data during the experimental period was monitored using the HOBO weather station (Onset Computer, Boume, Massachusetts, USA). The air temperature was measured using a shielded thermocouple at 10 cm above the canopy. The soil moisture was measured using an ECH2O™ EC5 soil moisture sensor placed at a soil depth of 45 cm. The rainfall was recorded using a tipping bucket mechanism with a resolution of 0.2 mm.

The data on chlorophyll index (SPAD units), the minimum chlorophyll fluorescence level (F_o_; dimensionless), the maximum quantum yield of photosystem II (F_v_/F_m_ ratio), gas exchange, stomatal movement, relative water content (%), leaf water potential (MPa), hydrogen peroxide (mmol g^−1^), malondialdehyde (mmol g^−1^), catalase (mmol H_2_O_2_ min^−1^ g^−1^), and peroxidase (mmol H_2_O_2_ min^−1^ g^−1^) enzymes activity were recorded as detailed below in the top fully expanded and penultimate leaf on 4^th^, 6^th^, and 8^th^ days after stress imposition, except leaf water potential. On the 8^th^ day after stress imposition, the leaf water potential was recorded.

The leaf chlorophyll index was recorded non-destructively using a chlorophyll meter, and chlorophyll *a* fluorescence, was measured with a modulated fluorometer (OS5p+, Opti Sciences, Hudson, NH), as detailed by Djanaguiraman et al. (2024). Canopy temperature was measured using an infrared thermal imager (Raytek, Wilmington, NC, USA) and expressed in °C. Gas exchange traits were measured using an infrared gas analyzer (CI-340 portable photosynthesis system, CID Inc., Camas, WA, USA), as described by Djanaguiraman et al. (2024). The leaf was allowed to habituate for 5 min before measurements began, and measurements were taken with the leaves oriented perpendicular to the sun, with the PPFD reaching the leaf surface ≥1,400 μmol m^−2^ s^−1^. Atmospheric CO_2_ concentration (410 μmol mol^−1^) was maintained inside the leaf chamber, and the measurements were recorded at ambient relative humidity and temperature.

Relative water content (RWC) was estimated following the procedure of Barrs and Weatherley (Barr and Weatherley, 1962) and expressed as a percentage. The leaf water potential (Ψ_w_) was measured using a pressure bomb apparatus (ARIMAD 3000, MRC lab, Essex, UK). The third leaf from the top of each replicate was excised, and the leaf base was cut using a sharp razor. The leaf was rolled and immediately inserted into the chamber, and the lid was tightened. Pressure was applied gradually until the water was oozed out from the cut surface of the leaf. The pressure at that moment was recorded as the tissue water potential and was expressed as -MPa.

The H_2_O_2_ was quantified using the procedure described by Patterson et al. (1984) and expressed in nmol g^−1^. Lipid peroxidation (MDA) was quantified as per Behera et al. (1999). The CAT enzyme activity was quantified according to the method described by Samantary (2002) and expressed in mmol H_2_O_2_ destroyed min^−1^ g^−1^. The POX enzyme activity was quantified according to Lin and Kao (1999) and expressed in mmol tetraguaiacol formed min^−1^ g^−1^. The detailed methodology of H_2_O_2_ estimation and CAT and POX enzyme activities was described by Djanaguiraman et al. (2024).

At physiological maturity, the plants per m^−2^ were harvested, separated into various parts, and dried at 60 °C for 48 h. Dry weight was measured and expressed as the total dry matter production (kg ha^−1^). The cob was cleaned, and the seeds were collected and expressed as number of seeds cob^−1^. Seed weight was expressed as seed yield (kg ha^−1^). The harvest index was calculated as the ratio between grain yield and total dry matter production and was expressed as a percentage (Djanaguiraman et al., 2014).

### 2.5 Statistical analysis

The earthworm and azolla toxicity experiment was designed in a completely randomized block design, and the data were analyzed using a four-parameter logistic regression model, as it provided the highest *r* ^2^ and smaller root mean square error (RSME). The normalized transpiration rate (NTR) was calculated as described by Sinclair and Ludlow (1986). In the progressive soil drying experiment, the relationship between transpiration rate and FTSW, transpiration rate, and time of day was analyzed using a broken-stick model, as this provided the highest *r* ^2^ and a smaller RSME. The pot culture experiment was conducted in a factorial randomized block design, and the data were analyzed using SAS PROC GLM. The field experiment was conducted using a randomized block design and the data were analyzed using the SAS PROC GLM model. Replications were considered blocks during statistical analysis. Separate and pooled data were analyzed, and the results from each day, individually or in combination, showed similar responses and significance for all traits. Therefore, the mean response across the day of observation was presented. Mean separation was performed using the Tukey–Kramer adjustment method.

## 3 Results

### 3.1 Characterization of ZnSe QDs

**Figure 1:**
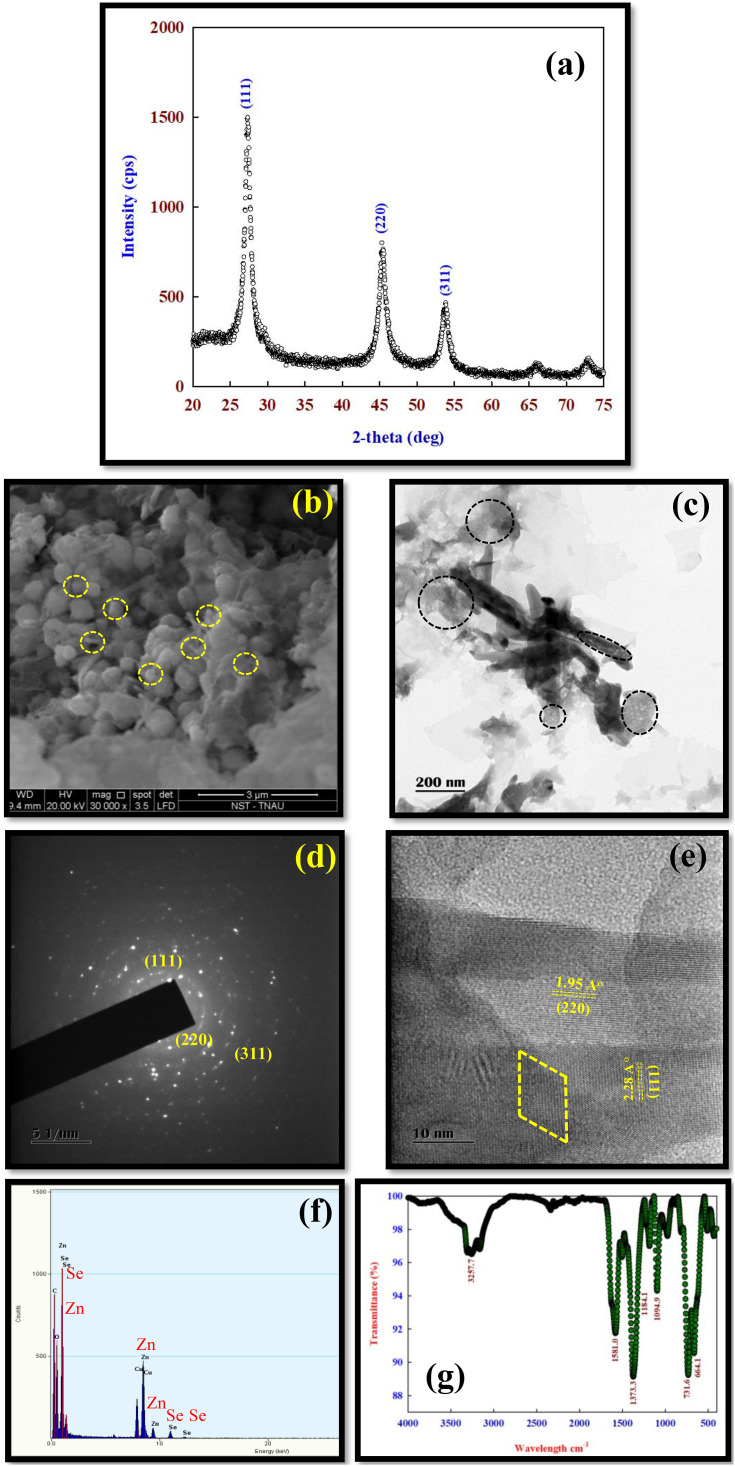
The characterization of ZnSe samples. **(A)** XRD pattern for ZnSe particles showing the (111), (220), and (311) planes corresponding to 2θ value of 27.2, 45.3, and 53.6, which was a cubic zinc blende structure (JCPDS card no: 80-0021). **(B)** The representative scanning electron microscope (SEM) image showing spherical particles with an average particle size of <10 nm. **(C)** The representative transmission electron microscope (TEM) image showing spherical particles with a particle size of <10 nm. **(D)** The selected area electron diffraction (SAED) showed a quasi-ring-like diffraction pattern with several dots indicating that ZnSe was a nanocrystallite. The high-resolution transmission electron microscope (HRTEM) diffraction pattern showed (111), (220), and (311) reflection, which corresponds to XRD data. **(E)** The interplanar spacing was 0.32 and 0.21 nm, which corresponds to the (111) and (220) plane, respectively, of the cubic structure of ZnSe, which agrees with XRD. **(F)** The composition of the ZnSe nanoparticle was determined by the energy dispersive analysis of X-rays (EDAX), and the particle did not contain impurities. **(G)** The Fourier transform Infrared (FT-IR) spectrum of ZnSe showed the dominant peaks at 3257 cm^-1^, 1581 cm^-1^, and 1373 cm^-1^.

The XRD pattern of ZnSe showed three major peaks at 2θ values of 27.2, 45.3, and 53.6, which correspond to the (111), (220), and (311) diffraction planes, respectively (**Figure 1A**). The average diameter ZnSe was calculated using the Debye–Scherrer formula based on the full width at half maximum of the (111) peak and was 4.2 nm. The SEM image of the ZnSe nanoparticles showed that they were spherical and aggregated, with an average particle size of 10 nm–20 nm (**Figure 1B**).

The HRTEM image showed that ZnSe was spherical with a particle size of <10 nm (**Figure 1C**). The SAED pattern of ZnSe showed a quasi-ring-like diffraction pattern with several spots, indicating the crystalline nature of ZnSe at the nanometer scale (**Figure 1D**). The interplanar spacing of ZnSe was 0.32 nm and 0.21 nm, corresponding to the (111) and (220) planes, respectively. This confirmed the cubic structure of the ZnSe phase, which was in good agreement with the XRD data (**Figure 1E**). The EDX spectrum confirmed the presence of Zn and Se without any impurities (**Figure 1F**). The FTIR spectra of ZnSe showed broad peaks at 3,257 cm^−1^, 1,581 cm^−1^, 1,373 cm^−1^, 664 cm^−1^, and 731 cm^−1^ (**Figure 1G**).

### 3.2 ZnSe QD toxicity

**Figure 2:**
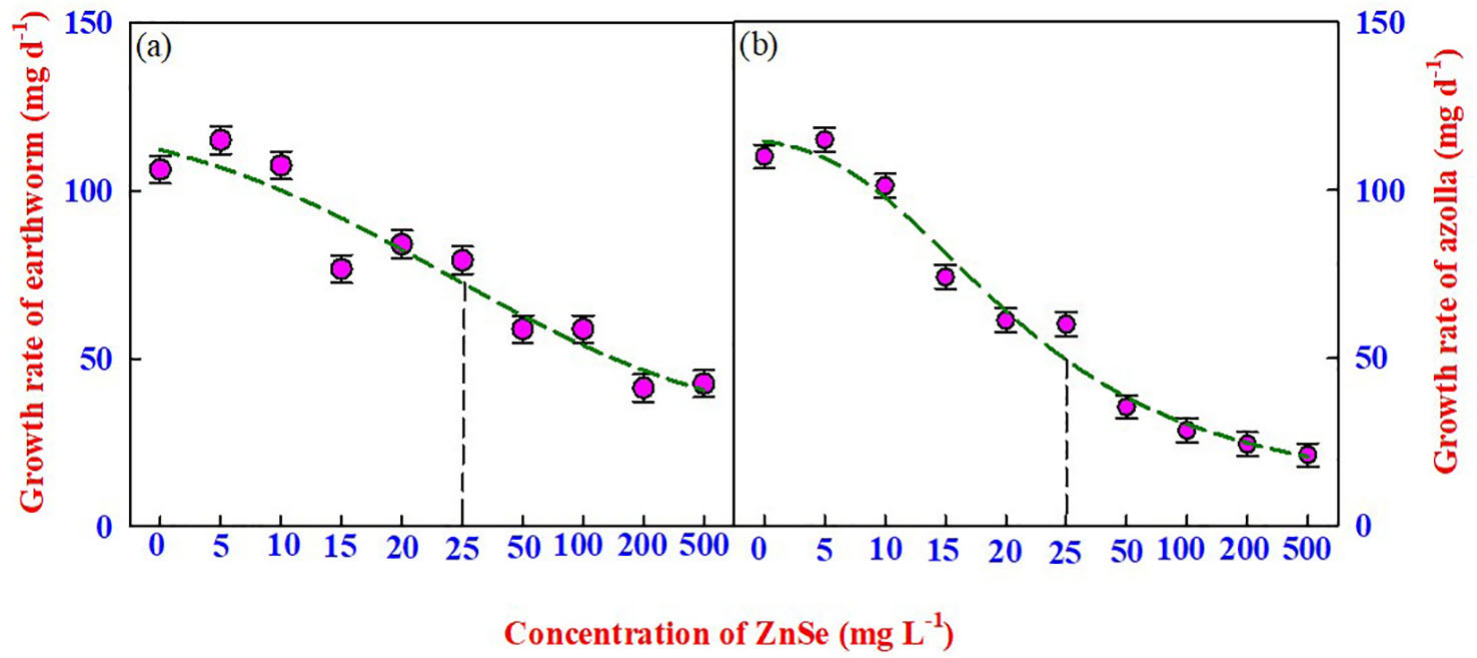
The effects of various concentrations of ZnSe (0 to 500 mg L^-1^) on the growth rate of **(A)** earthworms (*Eisenia Andrei)* and **(B)** azolla. The data are presented as mean ± standard error of the mean (n = 10). The vertical dotted line represents the LD_50_ value arrived at by a four-parameter logistic regression model.

The most appropriate method for assessing the toxicity of nanomaterials is to assess the mortality and body mass of earthworms over time. In this experiment, the survival of earthworms was recorded for 14 d in natural soil spiked with ZnSe QDs, and the results indicated no mortality up to 500 mg L^−1^ (**Figure 2A**). However, the earthworm growth rate (mg d^-1^) started to decrease significantly (P <0.05) from 0 mg L^−1^ to 15 mg L^−1^ (**Figure 2A**), and the LD_50_ concentration was from 25 mg L^−1^ of ZnSe QDs.

Similar to earthworms, azolla survived up to 500 mg L^−1^ (**Figure 2B**). However, the growth rate decreased significantly (P <0.05) from 0 mg L^−1^ to 10 mg L^−1^ (**Figure 2B**), and the LD_50_ concentration was from 25 mg L^−1^ for the ZnSe QDs. A greater decrease in the growth rate was observed at high concentrations (200 mg L^−1^ –500 mg L^−1^) of the ZnSe QDs.

### 3.3 Effects of ZnSe QDs under progressive soil drying

**Figure 3:**
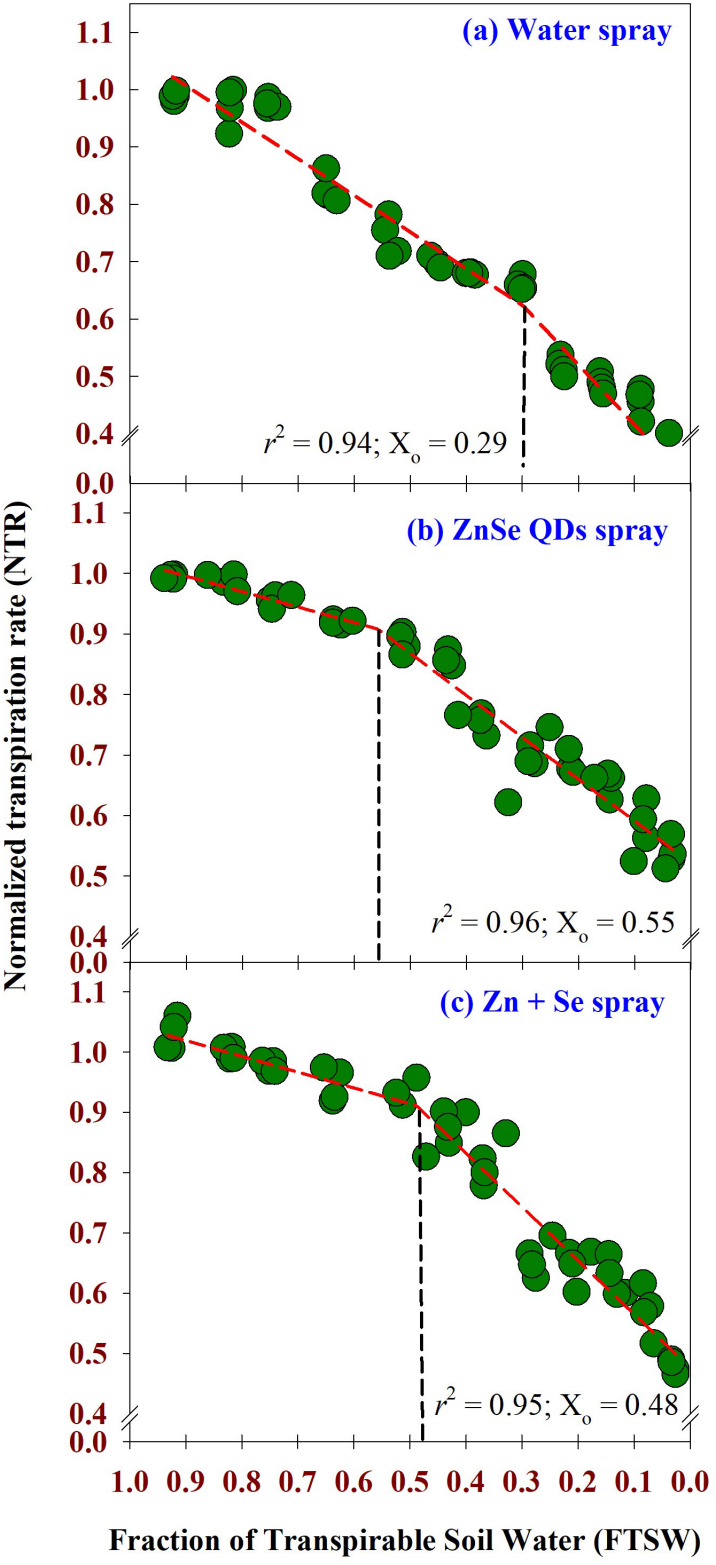
Normalized Transpiration Rate (NTR) – Fraction of Transpirable Soil Water (FTSW) response curve calculated using two-segment linear regressions. **(A)** water spray, **(B)** zinc selenide quantum dots spray (ZnSe) (20 mg L^-1^), and **(C)** combined zinc sulphate (10 mg L^-1^) and sodium selenate (10 mg L^-1^) (Zn+Se) spray. The maize plants were maintained under progressive soil drying under glasshouse conditions. Values are transpiration data are from four replicated plants for each foliar spray at each FTSW condition measured for 13 days (n=4). The FTSW thresholds where transpiration initiated its decline were calculated using a broken stick analysis. The regression lines of the relationships between NTR and FTSW were drawn using the Phyton 3.11.8 version. The *r* ^2^ value (0.94 - 0.96) was very high for all the foliar treatments. The FTSW threshold values (X_o_) are shown as dotted vertical lines in **(A)** water, **(B)** ZnSe, and **(C)** Zn+Se. The X_o_ is the point from which the transpiration rate decreases linearly.

The weather parameters during the cropping season are shown in **Supplementary Figure S1**. The maximum daytime temperature during the experimental period was between 42 °C and 43 °C. Similarly, the minimum night temperature ranged from 21 °C to 24 °C. Daytime relative humidity ranged from 78% to 83%. Similarly, nighttime relative humidity was between 20% and 30%. Across the foliar sprays, two-segment linear regression fitted the data of FTSW and NTR with *r* ^2^ = 0.94, 0.96, and 0.95, respectively, for water, ZnSe QDs, and combined Zn and Se spray (**Figures 3A–C**). The value of NTR was approximately one when the soil was comparatively wet (FTSW value between 0.99 and 0.95). However, NTR decreased linearly from the breakpoint for transpiration (**Figures 3A–C**). The decrease in NTR in relation to FTSW was steep for the water spray (**Figure 3A**). However, the slope was lower for the ZnSe QDs (**Figure 3B**). The breakpoints for the transpiration rate for FTSW, ZnSe QDs and combined Zn and Se spray were respectively 0.29, 0.55, and 0.48.

**Figure 4:**
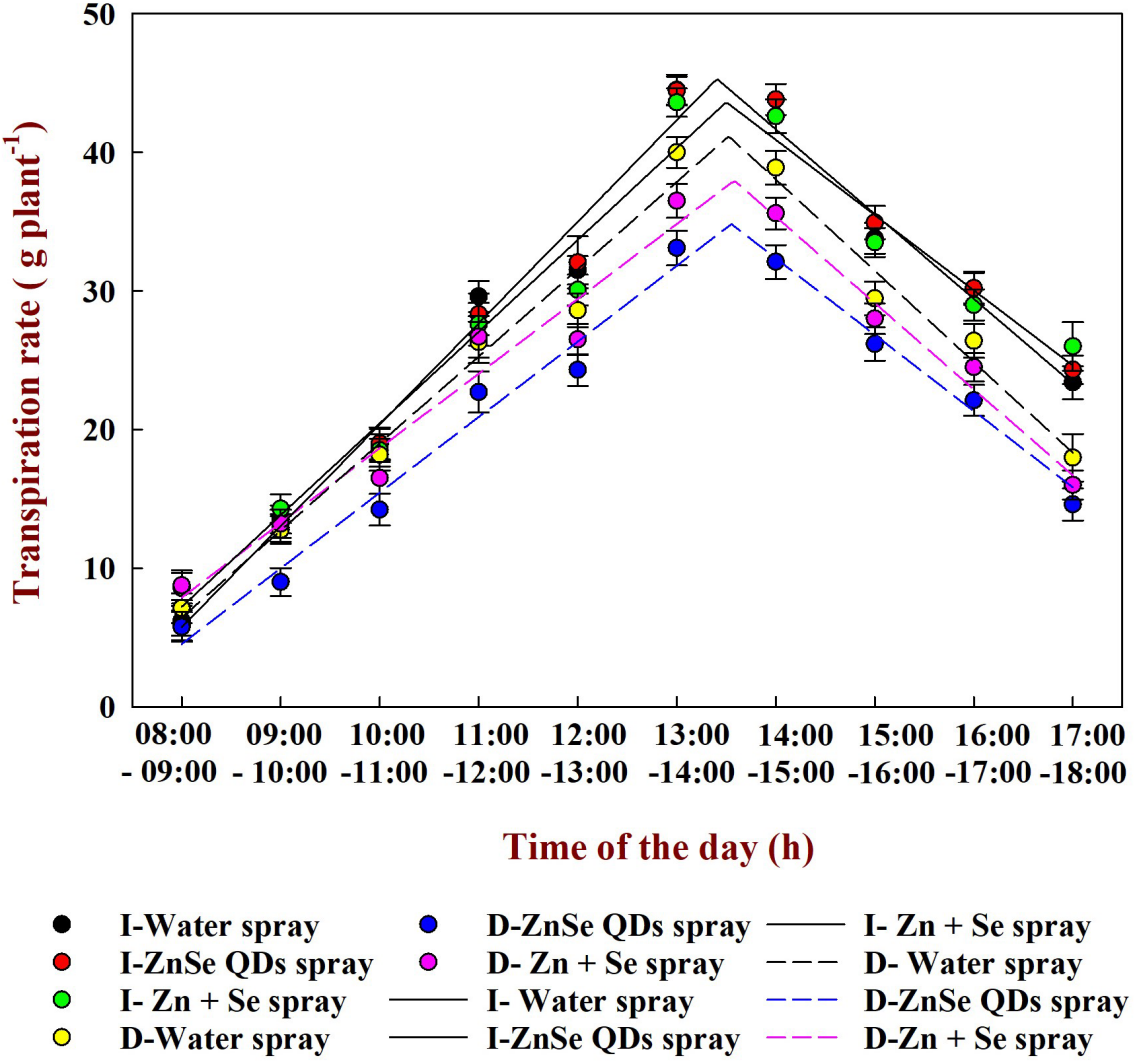
Daytime transpiration rate – Time of the day response curve calculated using two-segment linear regressions. The *r* ^2^ value (0.96 - 0.97) was very high for all the foliar treatments. The maize plants were maintained under progressive soil drying in a naturally changing atmospheric vapour pressure deficit, and the transpiration rate was measured from 8:00 to 18:00 hours. The daytime transpiration rate was measured from the 3^th^ day (~0.9 FTSW) to the 10^th^ day after progressive soil drying (~0.25 FTSW). The regression lines of the relationships between the daytime transpiration rate and the time of the day were drawn using the Phyton 3.11.8 version. Each point is the hourly mean across 7 d of the experiment ±SE (n=28; 7 days and 4 replications). Where, I-Water spray: irrigated and water sprayed, I- ZnSe QDs: irrigated and foliar application of 20 mg L^-1^ of the zinc selenide quantum dots, I-Zn+Se: irrigated and foliar application of 10 mg L^-1^ of ZnSO_4_ + 10 mg L^-1^ of sodium selenate, D-Water spray: drought-stressed and water sprayed, D-ZnSe QDs: drought-stressed and foliar application of 20 mg L^-1^ of the zinc selenide quantum dots, D-Zn+Se: drought-stressed and foliar application of 10 mg L^-1^ of ZnSO_4_ + 10 mg L^-1^ of sodium selenate. The solid regression line explains the irrigated conditions, and the dashed lines explain the drought-stressed conditions.

**Figure 5:**
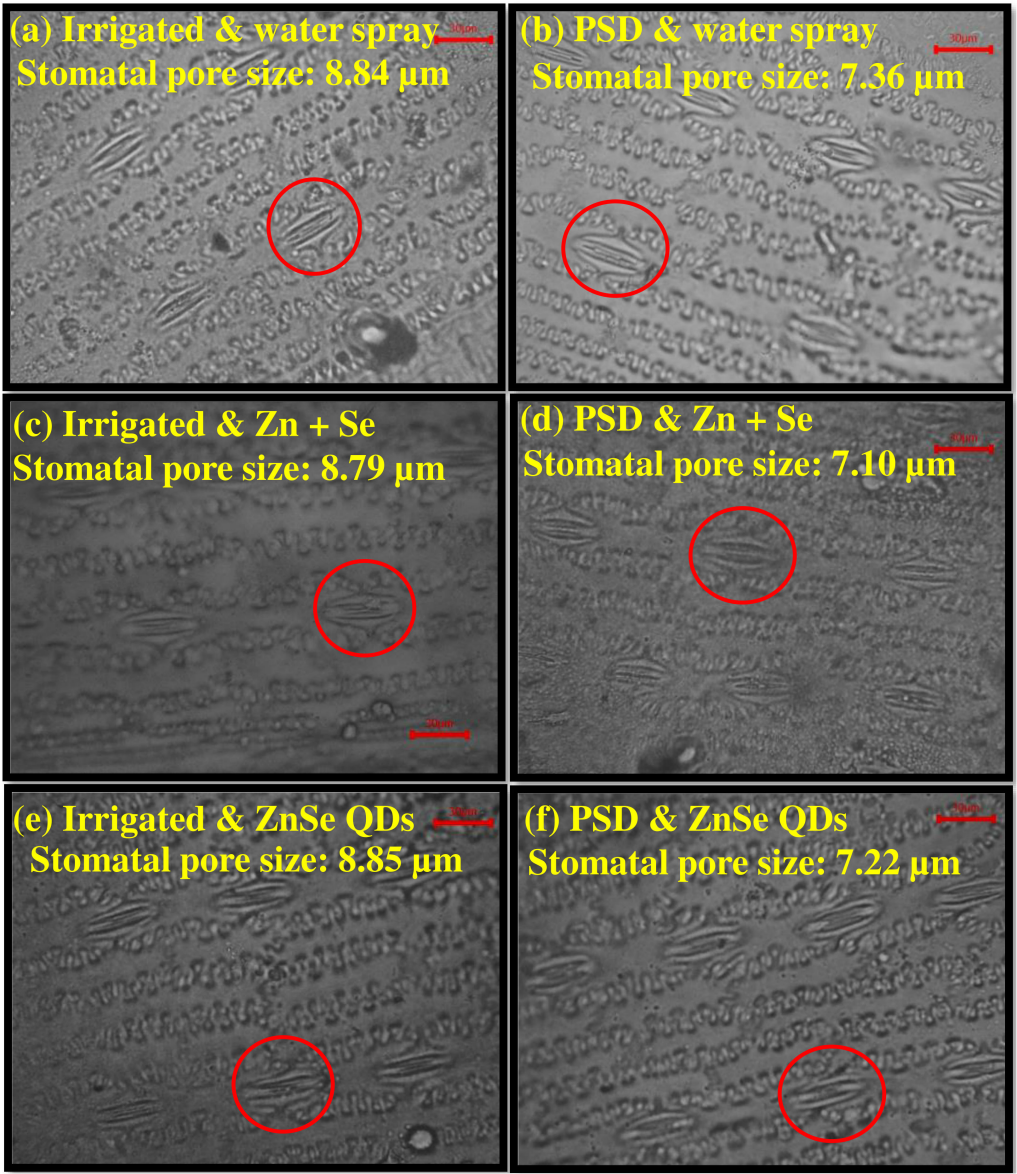
Representative images showing the effects of soil moisture and foliar sprays on the stomatal aperture. **(A)**, **(C)** and **(E)** are irrigated plants, and **(B)**, **(D)** and **(F)** are progressive soil drying plants (PSD). **(A)** and **(B)** water spray, **(C)** and **(D)** combined zinc sulphate (10 mg L^-1^) and sodium selenate (10 mg L^-1^) spray (Zn + Se), **(E)** and **(F)** zinc selenide quantum dots (ZnSe) spray (20 mg L^-1^). The pore diameter in each treatment is shown in the figure. All the treatment combinations had open stomata; however, plants under progressive soil drying (PSD) had partially closed stomata.

**Figure 6:**
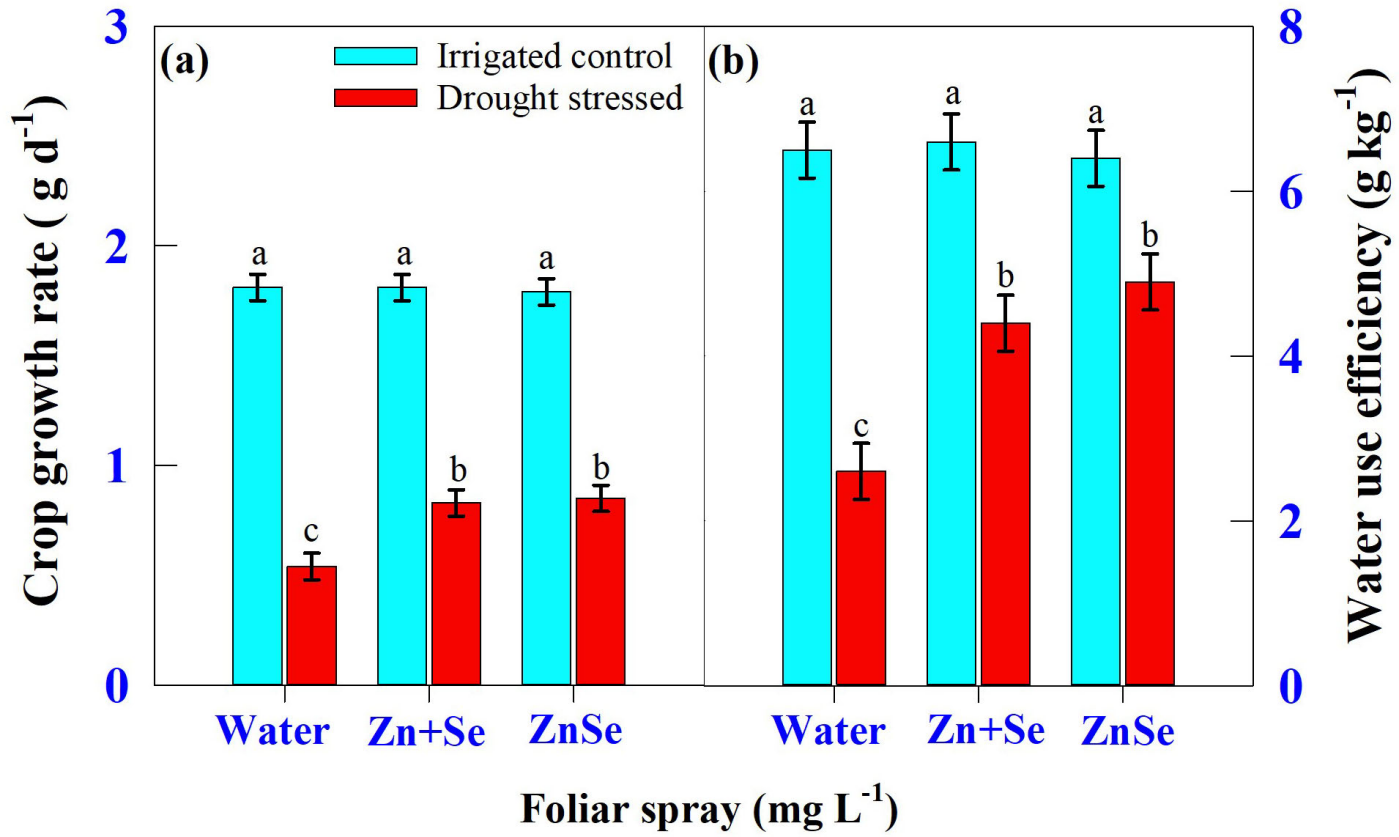
Interaction of soil moisture regime [irrigated control: watered every evening to maintain 0.9 fraction of transpirable water (FTSW) and drought-stressed: the plants were maintained under progressive soil drying (from 0.95 to 0.05 FTSW)] and foliar spray [water spray; combined zinc sulphate (10 mg L^-1^) and sodium selenate (10 mg L^-1^) spray (Zn + Se); and zinc selenide quantum dots (ZnSe) spray (20 mg L^-1^)] on **(A)** crop growth rate (g d^-1^) and **(B)** water use efficiency (g kg^-1^). Each datum was the mean ± standard error of the mean (n=4). Means with different letters are significantly different at P<0.05.

Seven days of daytime hourly transpiration data (g plant^−1^) indicated that plants under progressively drying soil had a significantly (P <0.05) reduced transpiration rate when compared to the irrigated control (**Figure 4**). Similarly, in all seven days under progressive drying, the plants sprayed with ZnSe QDs had a significantly reduced daytime hourly transpiration rate over the water spray (**Figure 4**). All the plants under irrigated control had a stomatal pore size between 8.79 µm and 8.85 µm (**Figures 5A, C, E**). However, under progressive soil drying, the stomatal pore size was between 7.10 µm and 7.36 µm (**Figures 5B, D, F**). Among the foliar sprays under progressive soil drying, ZnSe exhibited a small pore size of 7.10 µm. Data on the rate of dry matter accumulation (g day^−1^) and WUE (g kg^−1^) under progressive soil drying indicated that foliar application of ZnSe QDs increased the rate of dry matter accumulation (**Figure 6A**; 65%) and WUE (**Figure 6B**; 78%).

### 3.4 Effects of ZnSe under rainfed condition

#### 3.4.1 Environment

The weather parameters during the cropping season are shown in **Supplementary Figure S2**. The maximum daytime temperature during the experimental period was between 24 °C and 38 °C. Similarly, the nighttime minimum temperature ranged between 18 °C and 25 °C. The total amount of rainfall received during the cropping season was 5.6 mm. Up to the sixth leaf stage, the soil moisture content ranged between 0.58 m^3^ m^−3^ and 0.57 m^3^ m^−3^, started declining drastically and reached 0.2 m^3^ m^−3^ at harvest.

#### 3.4.2 Leaf tissue water

**Figure 7:**
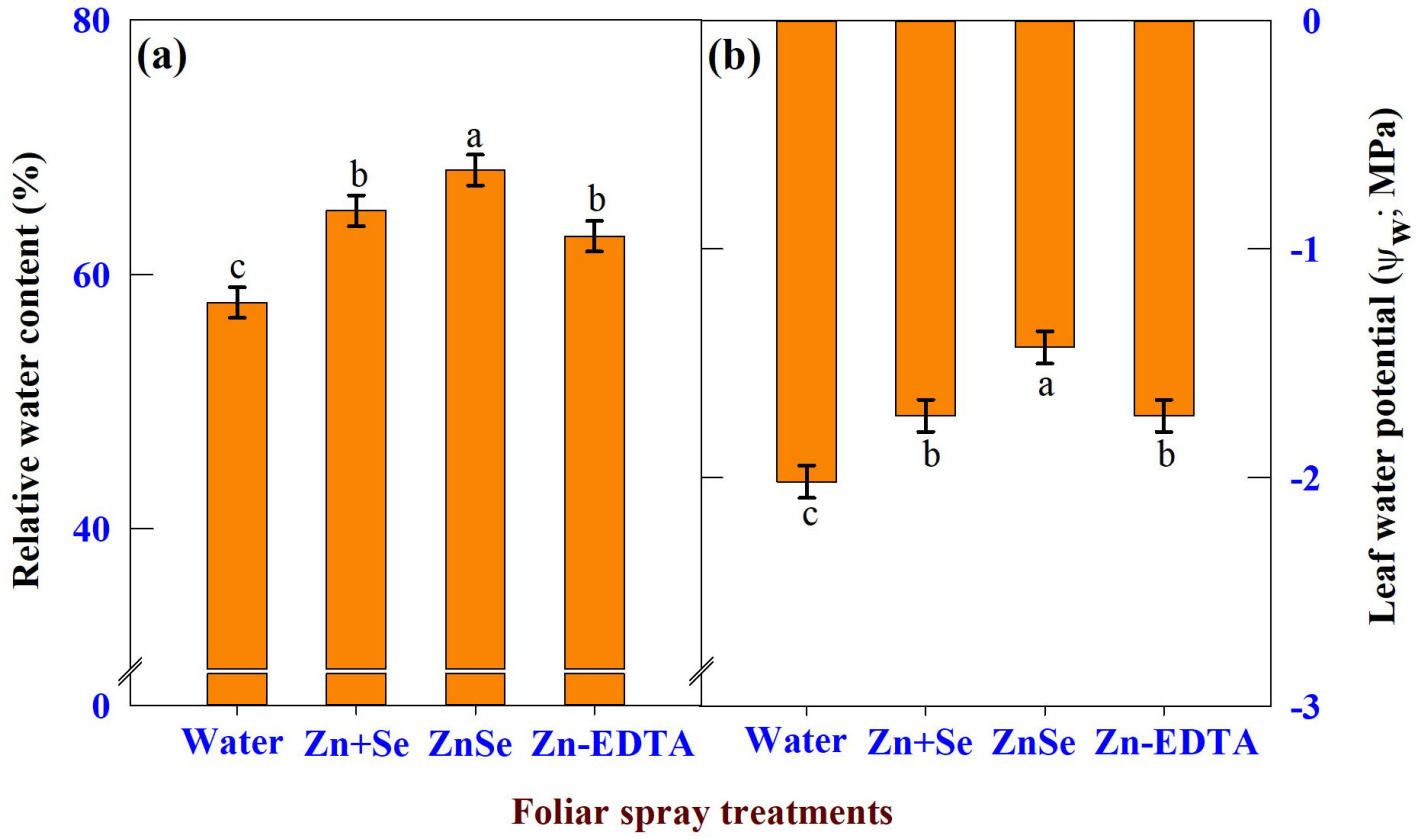
Effects of foliar spray [water spray; combined zinc sulphate (10 mg L^-1^) and sodium selenate (10 mg L^-1^) spray (Zn + Se); zinc selenide quantum dots (ZnSe) spray (20 mg L^-1^) and zinc-EDTA (Zn-EDTA)] on **(A)** relative water content (%), and **(B)** leaf water potential (Ψ_w_; MPa) of rainfed maize. The plants experienced drought stress from the tasselling stage to the physiological maturity stage (50 to 100 DAS). The data were recorded on the 52^nd^, 54^th^, and 56^th^ DAS. Each datum was the mean ± standard error of the mean (n=12; 3 times and 4 replications). Means with different letters are significantly different at P<0.05.

The effects of foliar spray were significant (P <0.05) for RWC (**Figure 7A**) and leaf water potential (**Figure 7B**). Foliar application of ZnSe QDs increased leaf RWC (17%) and leaf water potential (29%) compared to water spray. Similar to ZnSe QDs, foliar application of a combined zinc and selenium spray increased the RWC (12%) and decreased the leaf water potential (14%).

#### 3.4.3 Chlorophyll index and chlorophyll fluorescence

**Figure 8:**
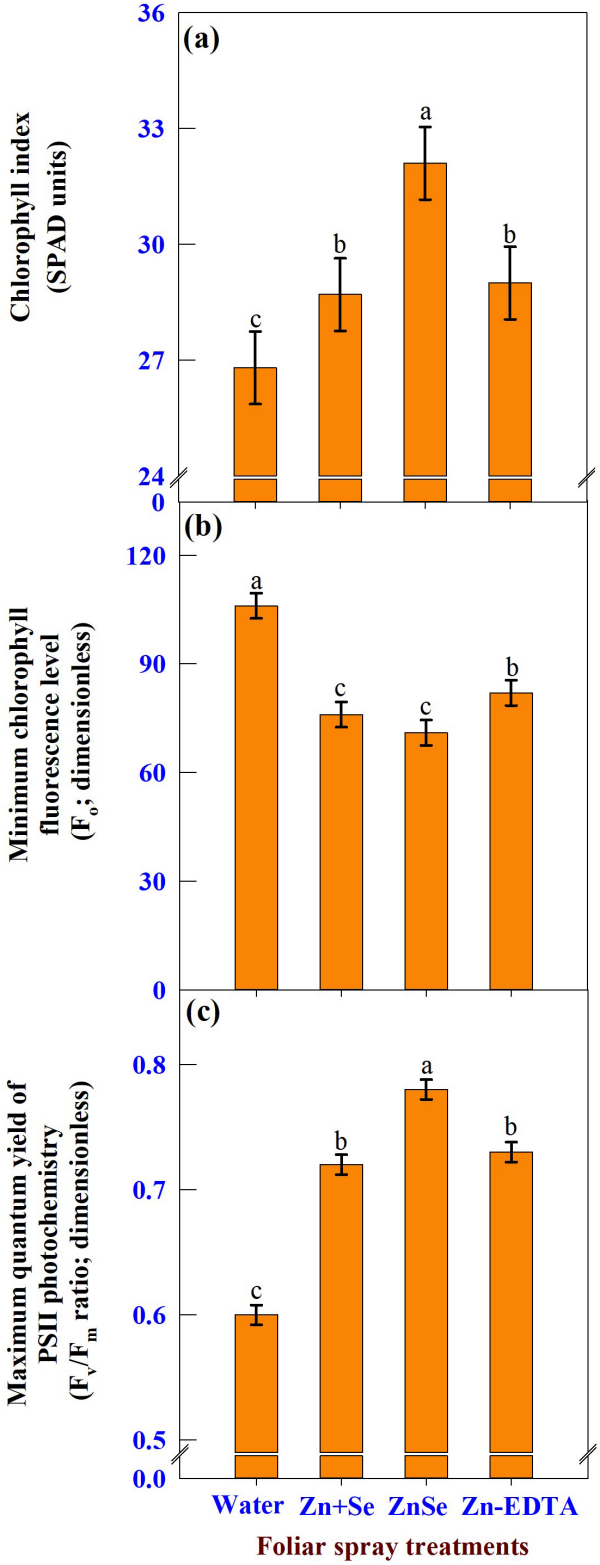
Effects of foliar spray [water spray; combined zinc sulphate (10 mg L^-1^) and sodium selenate (10 mg L^-1^) spray (Zn + Se); zinc selenide quantum dots (ZnSe) spray (20 mg L^-1^) and zinc-EDTA (Zn-EDTA)] on **(A)** chlorophyll index (SPAD units), **(B)** minimum chlorophyll fluorescence level (F_o_; dimensionless), and **(C)** maximum quantum yield of PS II photochemistry (F_v_/F_m_; dimensionless) of rainfed maize. The plants experienced drought stress from the tasselling stage to the physiological maturity stage (50 to 100 DAS). The data were recorded on the 52^nd^, 54^th^, and 56^th^ DAS. Each datum was the mean ± standard error of the mean (n=12; 3 times and 4 replications). Means with different letters are significantly different at P<0.05.

The effects of foliar spray were significant (P <0.05) for the chlorophyll index (**Figure 8A**, SPAD units), minimum chlorophyll fluorescence level (**Figure 8B**, F_o_; dimensionless), and maximum quantum yield of PS II photochemistry (**Figure 8C**, F_v_/F_m_ ratio; dimensionless). The application of ZnSe QDs at the tasselling stage increased the chlorophyll content (20%) and F_v_/F_m_ ratio (30%) compared with water spray. In contrast, the F_o_ value decreased by 29% compared that of the water spray.

#### 3.4.4 Gas exchange

**Figure 9:**
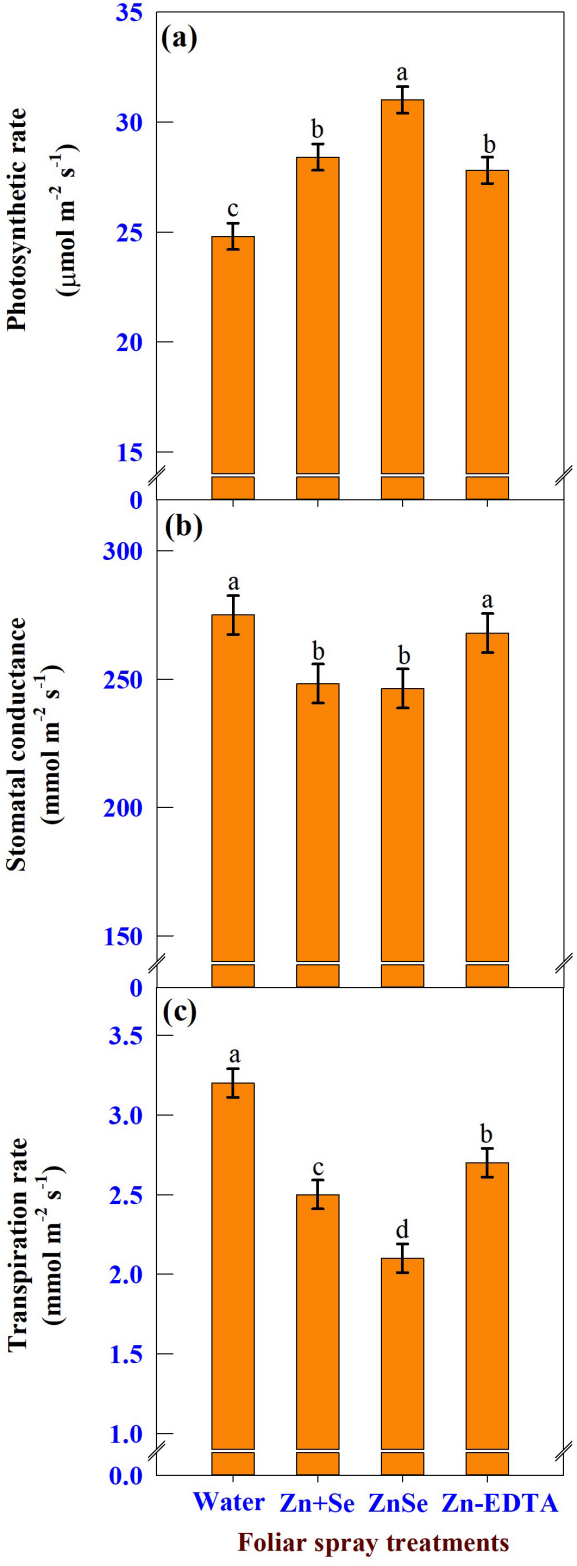
Effects of foliar spray [water spray; combined zinc sulphate (10 mg L^-1^) and sodium selenate (10 mg L^-1^) spray (Zn + Se); zinc selenide quantum dots (ZnSe) spray (20 mg L^-1^) and zinc-EDTA (Zn-EDTA)] on **(A)** photosynthetic rate (µmol m^-2^ s^-1^), **(B)** stomatal conductance (mmol m^-2^ s^-1^), and **(C)** transpiration rate (mmol m^-2^ s^-1^) of rainfed maize. The plants experienced drought stress from the tasselling stage to the physiological maturity stage (50 to 100 DAS). The data were recorded on the 52^nd^, 54^th^, and 56^th^ DAS. Each datum was the mean ± standard error of the mean (n=12; 3 times and 4 replications). Means with different letters are significantly different at P<0.05.

Under rainfed conditions, foliar application of ZnSe QDs, zinc and selenium, and water significantly (P <0.05) influenced photosynthetic rate (**Figure 9A**), stomatal conductance (**Figure 9B**), and transpiration rate (**Figure 9C**). The photosynthetic rate was increased by the application of ZnSe QDs (25%) application over the water spray. However, the stomatal conductance (10%) and transpiration rate (34%) were lower than those of the water spray. A similar response was observed with the foliar application of combined zinc and selenium.

#### 3.4.5 H_2_O_2_, MDA, CAT, and POX enzyme activity

**Figure 10:**
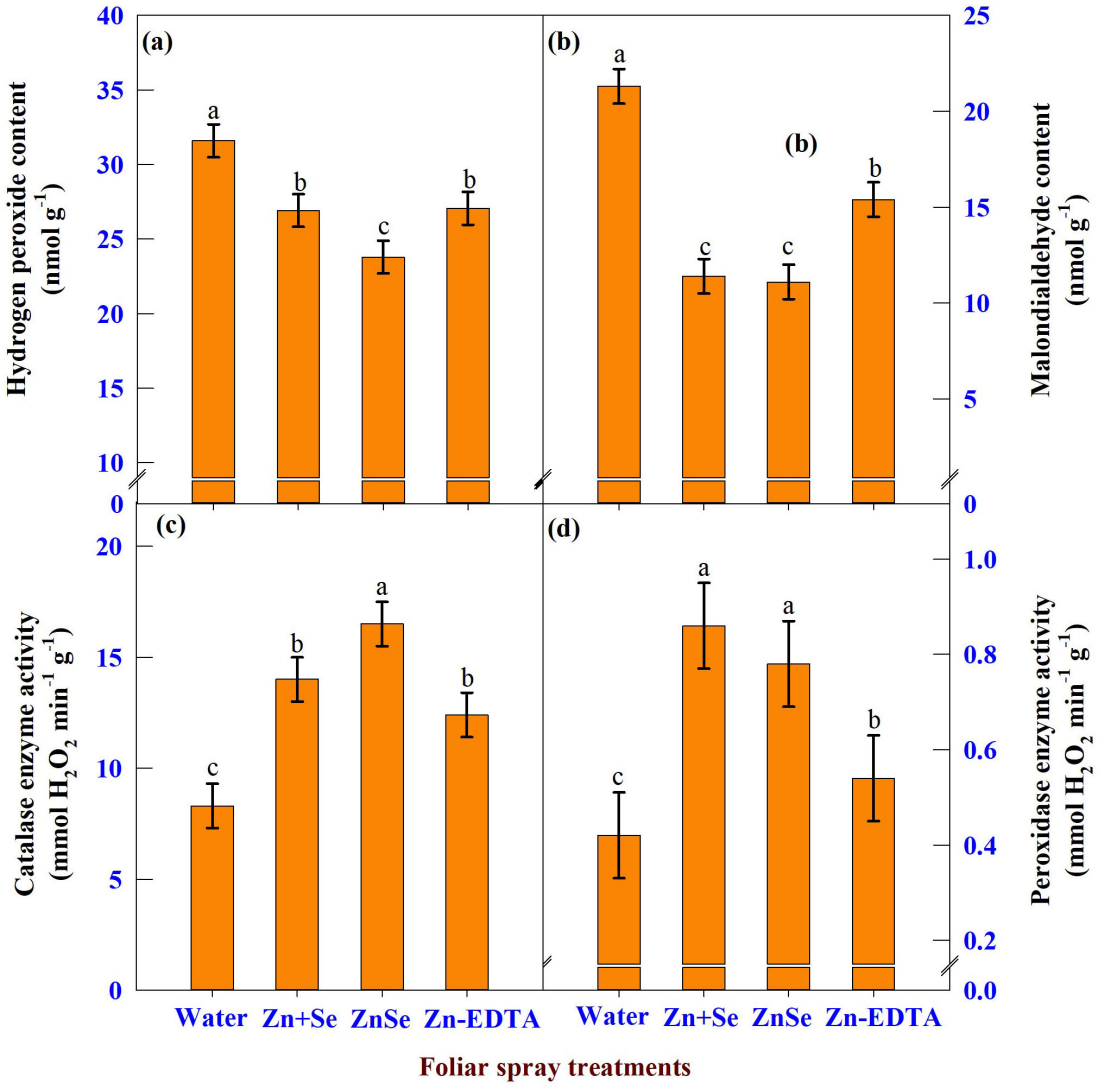
Effects of foliar spray [water spray; combined zinc sulphate (10 mg L^-1^) and sodium selenate (10 mg L^-1^) spray (Zn + Se); zinc selenide quantum dots (ZnSe) spray (20 mg L^-1^) and zinc-EDTA (Zn-EDTA)] on **(A)** hydrogen peroxide content (nmol g^-1^), **(B)** malondialdehyde content (nmol g^-1^), **(C)** catalase enzyme activity (mmol H_2_O_2_ destroyed min^-1^ g^-1^), and **(D)** peroxidase enzyme activity (mmol H_2_O_2_ destroyed min^-1^ g^-1^) of rainfed maize. The plants experienced drought stress from the tasselling stage to the physiological maturity stage (50 to 100 DAS). The data were recorded on the 52^nd^, 54^th^, and 56^th^ DAS. Each datum was the mean ± standard error of the mean (n=12; 3 times and 4 replications). Means with different letters are significantly different at P<0.05.

The data on H_2_O_2_ (**Figure 10A**), MDA (**Figure 10B**), CAT (**Figure 10C**), and POX (**Figure 10D**) enzyme activity showed significant (P <0.05) variation due to the application of ZnSe QDs, chemicals, and water. The application of ZnSe QDs decreased H_2_O_2_ (23%) and MDA (47%) compared with water spray. In contrast, the activities of CAT (98%) and POX (85%) increased over water spray. Similar to ZnSe QDs, the combination of zinc and selenium also reduced the oxidant content and increased the activity of antioxidant enzymes.

#### 3.4.6 Yield and yield components

The data on total dry matter production (kg ha^−1^), number of seeds row^−1^ cob^−1^, number of seeds cob^−1^, seed yield (kg ha^−1^), and harvest index (%) were significant (P <0.05) for foliar spray (**Table 1**). ZnSe QDs increased the total dry matter production (23%), number of seeds row^−1^ cob^−1^ (40%), number of seeds cob^−1^ (42%), seed yield (26%), and harvest index (5%) than water spray. Similarly, a foliar spray of combined zinc and selenium increased the total dry matter production (10%), number of seeds row^−1^ cob^−1^ (14%), number of seeds cob^−1^ (20%), seed yield (12%), and harvest index (3%) compared with water spray.

**Table 1:** Effects of foliar spray (water, bulk material (ZnSO_4_ @ 10 mg L^-1^ and sodium selenate @ 10 mg L^-1^), ZnSe QDs @ 20 mg L^-1^, and EDTA-zinc @ 20 mg L^-1^) on total dry matter production (kg ha^-1^), number of seeds row^-1^ cob^-1^, number of seeds cob^-1^, seed yield (kg ha^-1^) and harvest index (%) of maize plants grown under rainfed condition.

Treatment	Total dry matter production (kg ha^-1^)	Number of seeds row^-1^ cob^-1^	Number of seeds cob^-1^	Seed yield (kg ha^-1^)	Harvest index (%)
S_1_ - Water spray	4433.1^b^	20.50^c^	333.5^c^	2870.6^c^	30.86^a^
S_2_ - Bulk material (ZnSO_4_ @ 10 mg L^-1^ and sodium selenate @10 mg L^-1^)	4883.2^ab^	23.38^b^	402.5^b^	3237.6^b^	31.74^a^
S_3_ – ZnSe QDs @ 20 mg L^-1^	5466.6^a^	28.70^a^	474.0^a^	3633.6^a^	32.26^a^
S4 - Zn-EDTA @ 20 mg L^-1^	4999.9^ab^	24.33^b^	420.5^b^	3336.3^b^	32.06^a^
*C.D*	*592.7*	*2.87*	*51.6*	*292.5*	*5.15*

Within a column, means with same letter are non-significant at P<0.05.

## 4 Discussion

The major findings of this study are: (i) the synthesized ZnSe QDs were quasi-spherical with 10 nm, confirmed through XRD, SEM, and TEM, (ii) the exposure of earthworm and azolla to ZnSe QDs up to 20 mg L^−1^ did not cause any toxic effects, but, above this concentration, harmful effects occurred, (iii) foliar application of ZnSe @ 20 mg L^−1^ to maize plants grown under progressive soil drying restricted the transpiration rate at higher soil moisture level through partial stomatal closure compared to water spray, and (iv) application of ZnSe @ 20 mg L^−1^ to rainfed maize reduced leaf oxidative damage and increased photosynthetic rate and seed set percentage, resulting in higher seed yield.

The XRD pattern of the ZnSe QDs showed three major diffraction peaks at 2θ values of 27.2, 45.3, and 53.6, corresponding to the (111), (220), and (311) diffraction planes, respectively, which matched the cubic zinc-blende structure (Indirajith et al., 2014; JCPDS card: 80-0021). The broad diffraction peaks of the (111), (220), and (311) planes indicate the nanocrystalline size of ZnSe (**Figure 1A**). Based on XRD data, it is concluded that (i) capping with cysteine has not changed the nature, phase and crystallinity of ZnSe, (ii) ZnSe had an average diameter of ~4 nm, indicating strong confinement, (iii) in addition to ZnSe peaks there was no additional peaks associated with the formation of ZnO or Se phase.

The SEM image showed that the ZnSe QDs were spherical and aggregated with an average particle size of 10 nm–20 nm (**Figure 1B**). The agglomeration of the particles is due to their high crystallinity (Gupta et al., 2016). The formation of semi-spherical particles may be due to the strong binding affinity of n-acetyl cysteine to the ZnSe surface, leading to the reduction of surface energies at all surfaces, resulting in the formation of ZnSe QDs (Bain et al., 2015). Similarly, the TEM image indicated that the particle was spherical, and the particle size was between 5 nm and 6 nm (**Figure 1C**), which agrees with the XRD data.

The HRTEM image shows that the ZnSe QDs had a good crystalline nature, as evidenced by the SAED patterns. The ZnSe QDs showed a quasi-ring-like diffraction pattern with several spots, which explains the crystalline nature of ZnSe at the nanometer scale (**Figure 1D**) (Sridevi et al., 2022). The interplanar spacing of ZnSe was 0.32 nm and 0.21 nm, corresponding to the (111) and (220) plane, respectively, of the cubic structure of the ZnSe phase, which was in close agreement with XRD data (**Figure 1E**) (Verma et al., 2015).

The FTIR spectra of the ZnSe QDs showed a broad peak at 3,257 cm^−1^, which was assigned to the –OH stretching mode (**Figure 1G**) (Mahapatra et al., 2014). The peaks at 1,581 cm^−1^ and 1,373 cm^−1^ were associated with the C=O stretching vibration mode of ZnSe QDs (Sridevi et al., 2021), which indicated that the formed product had low water content and an acetyl group as a capping agent in the ZnSe QDs. The peaks at 664 cm^−1^ and 731 cm^−1^ were associated with ZnSe (Gupta et al., 2022). The absence of an intense symmetric stretching band at 2,548 cm^−1^ in ZnSe, which is a characteristic feature of pure n-acetyl cysteine, indicates that many deprotonated n-acetyl cysteine molecules capped the surface of ZnSe.

The survival of earthworms up to 14 d in natural soil spiked with ZnSe QDs showed no mortality up to 500 mg L^−1^, and the growth rate (mg day^−1^) started to decrease from 15 mg L^−1^ of ZnSe QDs. Similar results were observed by McShane et al. (2012) and Vidane et al. (2023), who reported that exposing earthworms to rutile TiO_2_ at up to 10,000 mg kg^−1^ did not result in mortality. In general, earthworms avoid digging holes for food in polluted soils (Jovana et al., 2014) and use the stored glycogen and proteins in the body (Ye et al., 2016). The decrease in body weight at higher concentrations of ZnSe (>25 mg L^−1^) might be due to starvation (**Figure 2A**; LD_50_). The decrease in the growth rate of azolla at higher concentrations (>25 mg L^−1^; **Figure 2B**) of ZnSe could be associated with a reduced photosynthetic rate (Oukarroum et al., 2013; Zarate-Cruz et al., 2016). A similar observation was made in *Arabidopsis* challenged with 100 mg L^−1^ ZnO (Landa et al., 2012).

Reduced transpiration rates by ZnSe QDs spray (**Figures 3B**; 4) indicated a restricted but continuous water flow that coordinates with stomatal movement to prevent desiccation (Sinclair, 2018). The decreased daytime hourly transpiration rate in ZnSe QDs and combined Zn and Se spray was due to decreased stomatal pore size (**Figures 5B, D, F**), indicating a positive relationship between the stomatal pore size and transpiration rate (Comstock, 2002). An early partial stomatal closure trait induced by ZnSe at high FTSW might have been linked to effective water use (Djanaguiraman et al., 2024). Increased water use under drought stress by nanoparticle foliar spray was observed in maize (Veroneze-Júnior et al., 2020), barley (*Hordeum sativum* distichum; Rajput et al., 2018), and sorghum [*Sorghum bicolor* L. (Moench); Djanaguiraman et al., 2024] and decreased transpiration rates resulted in increased WUE (**Figures 6A, B**). A similar observation was recorded in drought-stressed wheat sprayed with iron-oxide nanoparticles (Manzoor et al., 2023).

The importance of maintenaning tissue turgidity has been widely documented. In the present study, among the foliar sprays, the application of ZnSe QDs increased Ψw and RWC compared to water spray (**Figures 7A, B**), indicating that ZnSe QDs can alleviate drought stress by maintaining a higher tissue water content by increasing Ψw (Robbins and Dinneny, 2015). Foliar application of ZnSe and combined zinc and selenium under rainfed conditions decreased hydrogen peroxide content and malondialdehyde contents (**Figures 10A, B**). In contrast, the activities of the CAT and POX enzymes increased. Based on this observation, it could be concluded that ZnSe and Se acted as antioxidants by increasing the antioxidant enzymes (Tendenedzai et al., 2013) and/or due to their inherent property (Kuršvietienė et al., 2020).

The reduced F_o_ value in the ZnSe or Se treatments represents an efficient transfer of excitation energy from the antenna to the reaction centers, resulting in lower ROS production and more photochemistry (Genty et al., 1989). Furthermore, as shown by an increase in the F_v_/F_m_ ratio, the enhanced photosynthetic rate induced by ZnSe or selenium foliar spray under drought stress could reduce the photooxidative damage of PSII by efficiently scavenging the ROS generated in the chloroplasts during the light reaction to zinc (Cakmak, 2000) and Se (Djanaguiraman et al., 2010).

Plants sprayed with ZnSe QDs or Se showed increased photosynthetic rate and decreased stomatal conductance and transpiration rate (**Figures 9A–C**). In the present study, the inverse relationship between stomatal conductance and photosynthetic rate was due to differences in leaf water potential (O'Toole and Cruz, 1980). The loss of the photosynthetic rate in water-sprayed plants may be associated with increased premature leaf senescence under drought stress caused by higher ROS levels (Djanaguiraman et al., 2010; Honda et al., 2021). In contrast, plants sprayed with ZnSe or Se showed decreased ROS content and increased activity of antioxidant enzymes (**Figures 10A–D**), thereby, delaying premature leaf senescence might be delayed (Djanaguiraman et al., 2010).

In maize, drought stress during pollination reduces grain yield by 4% per day and can be up to 8%, depending on the intensity of drought stress (Nielsen, 2002). In the present study, drought stress during the reproductive stage reduced seed number cob^−1^ and grain yield (**Table 1**; Boyer and Westgate, 2004). However, foliar application of ZnSe or Se increased grain yield by improving seed cob^−1^, suggesting that reproductive success was improved by ZnSe or Se under drought stress (Tedeschini et al., 2015).

## 5 Conclusions

The synthesized ZnSe QDs were nanocrystalline and had a size of 4 nm to 10 nm in size. ZnSe QDs at concentrations >25 mg L^−1^ were toxic to earthworms and azolla. Here, we demonstrate for the first time that ZnSe QDs increase drought tolerance by restricting the transpiration rate through partial stomatal closure. The increased photosynthetic rate in rainfed maize sprayed with ZnSe QDs is associated with delayed leaf senescence caused by reduced oxidative damage. The increase in grain yield was due to increased reproductive success, as evidenced by the increased seed cob^−1^. Thus, foliar application of ZnSe at a rate of 20 mg L^−1^ to rainfed maize increased grain yield by increasing photosynthesis and reproductive traits. Overall, it is evident that foliar application of ZnSe QDs to drought-stressed maize plants activates the antioxidant defense system, resulting in the alleviation of drought-induced oxidative damage in leaves. However, additional research is needed to understand the long-term effects of ZnSe QDs on soil, aquatic, and terrestrial organisms before their large-scale adoption.

## Data Availability

The original contributions presented in the study are included in the article/**Supplementary Material**. Further inquiries can be directed to the corresponding author.
