# Supplementary material for: Improvement of maize drought tolerance by foliar application of zinc selenide quantum dots
Source: Front Plant Sci. 2024 Dec 3;15:1478654. doi: 10.3389/fpls.2024.1478654 (PMC11658264; doi:10.3389/fpls.2024.1478654)

Supplementary Material

# Supplementary Figure S1.

# The weather conditions during the progressive soil drying.


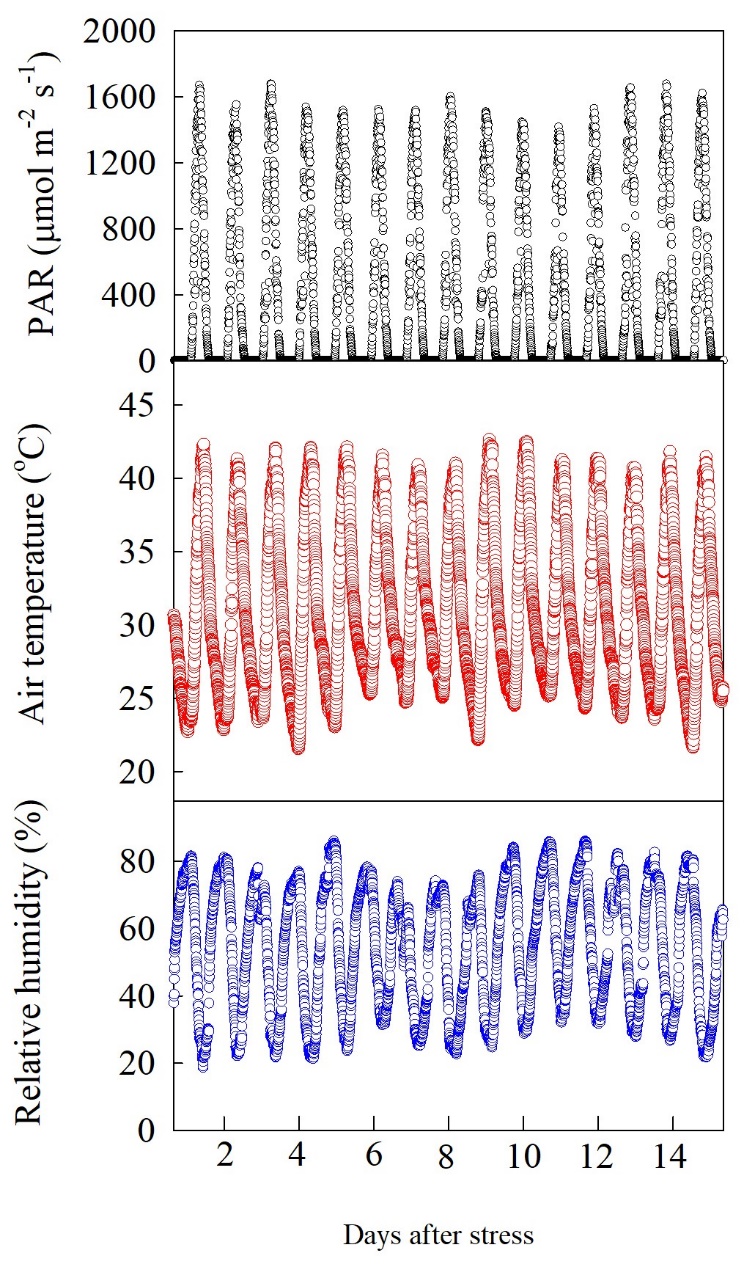


# Supplementary Figure S2.

# The weather conditions during the crop growing period of rainfed maize.


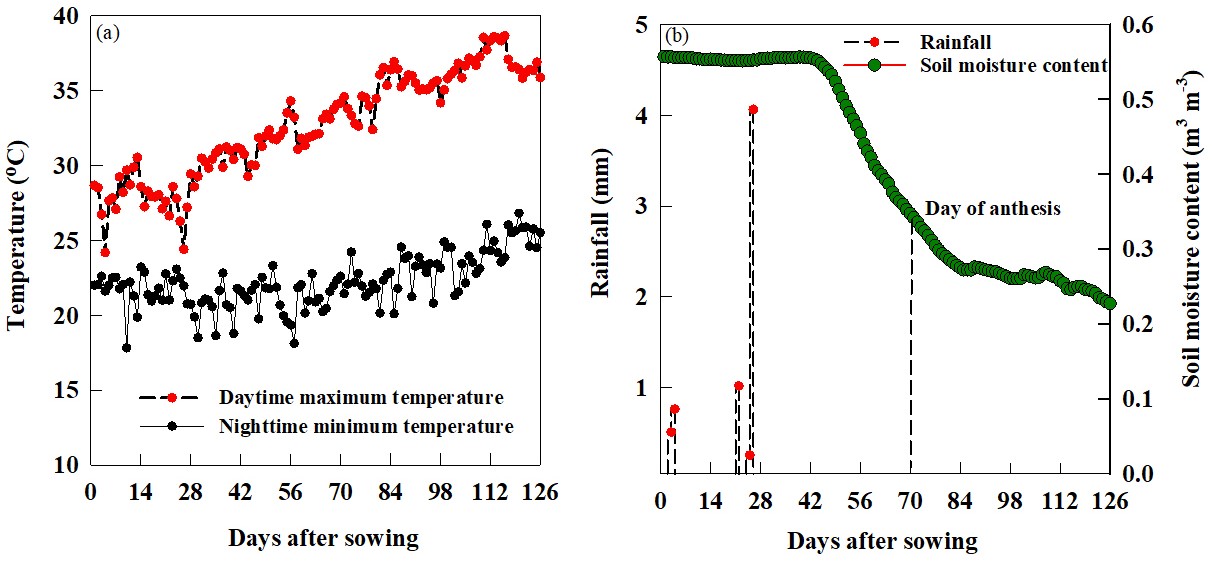

Supplement: Supplementary file 1 [file DataSheet1.docx]
